# Chair-Time During Polishing with Different Burs and Drills After Cement Customized Brackets Bonding: An In Vitro Comparative Study

**DOI:** 10.3390/dj13080347

**Published:** 2025-07-28

**Authors:** Javier Flores-Fraile, Alba Belanche Monterde, Oscar Alonso-Ezpeleta, Cosimo Galletti, Álvaro Zubizarreta-Macho

**Affiliations:** 1 Department of Surgery, Faculty of Medicine and Dentistry, University of Salamanca, 37008 Salamanca, Spain; j.flores@usal.es (J.F.-F.); amacho@uax.es (Á.Z.-M.); 2 Department of Obstetrics, Faculty of Health Sciences and Sports, University of Zaragoza, 20002 Huesca, Spain; lalonezp@unizar.es; 3 School of Medicine and Surgery Kore, University of Enna, 94100 Enna, Italy; cosimo.galletti@unikore.it; 4 Department of Dentisty, Faculty of Dentistry, Alfonso X University, Villanueva de la Cañada, 128091 Madrid, Spain

**Keywords:** tungsten carbide bur, efficiency, polishing, chair-time, customized bracket

## Abstract

**Introduction:** Digital planning and evolution of technology is allowing dentistry to be more efficient in time than before. In orthodontics the main purpose is to obtain fewer patient visits and to reduce the bonding time. For that, indirect bonding planned with CAD-CAM softwares is used to obtain a shorter treatment period, in general, and less chair-time. This waste of chair-time should also be reduced in other fields of dentistry such as endodontics, surgery, prosthodontics, and aesthetics. **Methods**: A total of 504 teeth were embedded into epoxy resin models mounted as a dental arch. Customized lingual multibracket appliances were bonded by a current adhesion protocol. After that, they were debonded, the polishing of cement remnants was performed with three different burs and two drills. The polishing time of each group was recorded by an iPhone 14 chronometer. **Results:** Descriptive and comparative statistical analyses were performed with the different study groups. Statistical differences (*p* < 0.005) between diamond bur and tungsten carbide and white stone burs and turbine were obtained, with the first being the slowest of them. **Discussion:** Enamel roughness was widely studied in orthodontics polishing protocol as the main variable for protocols establishment. However, in lingual orthodontics, due the difficulty of the access to the enamel surfaces, the protocol is not clear and efficiency should be considered. It was observed that the tungsten carbide bur is the safest bur. It was also recommended that a two-step protocol of polishing by tungsten carbide bur be followed by polishers. **Conclusions**: A tungsten carbide bur mounted in a turbine was the most efficient protocol for polishing after lingual bracket debonding.

## 1. Introduction

The polishing procedure is a concern in orthodontics due the possibility of damaging the enamel during the cement remnants clean-up. The polishing procedure can be time-wasting and challenging for the practician. The polishing protocol has been performed with different tools, such as diamond bur, tungsten carbide burs, or fiber-reinforced burs after brackets debonding [1]. In addition, due to the emergence of three-dimensional printing materials and different orthodontics systems, the polishing procedure has not been standardized [2]. The surface roughness after the polishing protocol has been a concern in this field because it produces biofilm retention and demineralization, so there is a higher risk of caries development. It has been observed that the superficial aprismatic enamel has a higher concentration of fluoride. Subsequently, excess polishing can produce damage to the superficial enamel, decreasing its resistance against acid bacterial products [3]. However, it has been observed that inevitable damage is produced on enamel after the debonding and polishing [2,3]. In addition, the increase in the amount of adult orthodontic patients has made the bonding brackets on prothesic surfaces necessary. As a consequence, the roughness on these surfaces after debonding and polishing has been studied with profilometry. It has been observed that diamond bur is efficient in decreasing surface roughness in disilicate, zirconia, and feldespathic but not in ceramics [4]. On the other hand, remineralizing agents are presented as a technology for ion release after bracket debonding and polishing in order to revert the enamel surfaces to the original state. Surface pre-reacted glass-ionomer (S-PRG) paste polishing has significantly recovered the mechanical proprieties of the enamel showing interesting outcomes [5]. Another remineralizing agent that has been studied is calcium-phosphate etchant paste. It seems that the preconditioning with this paste minimizes enamel damage by CaP reprecipitation and improves the efficiency of the polishing [6]. In addition, the debonding procedure is dependent on the shear bond strength (SB) the bracket has to the surface. It seems that SBS is dependent on the type of material in which the bracket is used. Ceramic brackets have reported higher damage rates of up to 63%; whereas, the modification of the bracket base seems to decrease this rate similar to metal brackets [7]. The bracket base individualization is a technique that emerged to reduce the side effects of the orthodontics mechanics and achieve a better fitting between the bracket base and the surfaces. SBS is being studied in three-dimensional printed individualized brackets, but the results show that improvements in the base design are needed. The individualization can be performed by modifying the metal of the bracket base or with a resin customization of the base [8]. In addition, lingual multibracket orthodontics have a particular biomechanics due to the interbracket distance being lower and the application of the forces made to the lingual surface of the crown being generally closer to the resistance center. As a consequence, the finalization phase might be more difficult because torque, tip, and rotational outcomes are more dependent of bonding precision. Also, the lack of homogenization of the lingual surfaces convexities makes the bonding technique more difficult in lingual orthodontics than in vestibular. So, currently lingual orthodontics brackets are individualized and indirectly bonded. The individualization of the base can be performed by adding compact resin to the metal base with the aid of manual or virtual set-ups [9,10]. Moreover, the indirect bonding decreases the overall transfer errors in the three planes of space. The silicone trays for indirect bonding have been recommended to obtain the highest accuracy [11]. The differences in polishing protocol using cement individualized and indirect bonded lingual bracket have been previously studied, but the evidence is still low [12].

The aim of the present study is to compare the chair-time between the polishing with diamond bur, white stone, and tungsten carbide bur with turbine and contra angle after customized lingual bracket debonding.

## 2. Methodology

### 2.1. Trial Design

This research is an experimental in vitro study performed at Salamanca University (Department of Surgery, Salamanca, Spain) between the years 2022 and 2024. In the research, indirect bonding brackets were used. The bracket system was specifically selected because it is a cement customized bracket bonded in lingual enamel. ALIAS brackets were bonded in 504 extracted teeth. A total of 144 incisors, 72 canines, 144 premolars, and 144 molars were used for the research in order to simulate a real dental arch. The teeth were embedded into epoxy resin simulating a dental arch, and the lingual brackets were bonded and debonded. After that, intraoral scanning was performed to evaluate the cement remnants, allowing us to measure it in volume and area [13]. The polishing protocol was performed without magnification. This study performed in accordance with the criteria defined in the regulations of the German Ethics Committee on the use of organic tissues in medical research (Zentrale Ethikkommission, 2003). The patients gave up the teeth after signing an Informed Consent.

### 2.2. Sample Size and Inclusion Criteria

Teeth were selected by enamel condition and anatomy. Teeth with restorations, prosthesis, or caries were excluded, and only teeth extracted because of orthodontics or periodontics reasons were included in the trial. In addition, microdontic or anomalous teeth were excluded. A total of 504 extracted teeth were included in the trial. The patients were correctly informed about the trial and an Informed Consent was signed. Power analysis was not performed, but the sample size was calculated based on similar articles sample sizes. In vitro trials about polishing after bracket debonding used n = 60 extracted teeth, so in this case a greater number than this size wanted to be used for more strong results [4]. In this trial, a total of n = 84 teeth per study group was used.

### 2.3. Sample Processes

Teeth were introduced into an epoxy resin model simulating a dental arch of 14 teeth and all segment teeth were included to create a current real arch. The teeth were randomized (Epidat 1.4, Galicia, Spain) for each study group by a second operator to reduce the risk of biases.

### 2.4. Interventions

Cement customized lingual brackets (ALIAS^TM^, Ormco, Orange, CA, USA) were indirectly bonded on the lingual surface of the teeth. The bonding protocol was a current protocol of adhesion in orthodontics. Etching with 37% orthophosphoric acid (Ortho Solo^TM^, Ormco Corporation, Orange, CA, USA) was performed on the lingual surfaces followed by water rinsing and a correct drying of the surfaces. After that, an adhesive primer (Unitek Transbond^TM^ XT, 3M ESPE^TM^, Saint Paul, MN, USA) was used, rinsed, and 10 s photopolymerized. The lingual brackets were correctly introduced into the positioning tray of indirect bonding, which was included into the packaging of an ALIAS system for each arch. Afterwards, a current orthodontic cement (BluePhase G2^TM^, Ivoclar Vivadent, Schaan, Principado de Liechtenstein) for braces was placed over each bracket base. The indirect tray was correctly placed on the epoxy resin model ensuring the fitting. Then a photopolymerization of each bracket was performed for 40 s. The tray was removed, and the brackets were correctly placed and bonded in each tooth. Cement remnants were removed with a bur. For that, 168 teeth were polished with tungsten carbide bur, 168 with diamond bur, and 168 with white stone bur. The diamond bur used was a particular bur for polishing with pear morphology and fine diamond grain. Also, these groups were separated into two for low-speed and high-speed hand pieces.

### 2.5. Methodology of Superimposition and Surface Assessment

**Figure 1:**
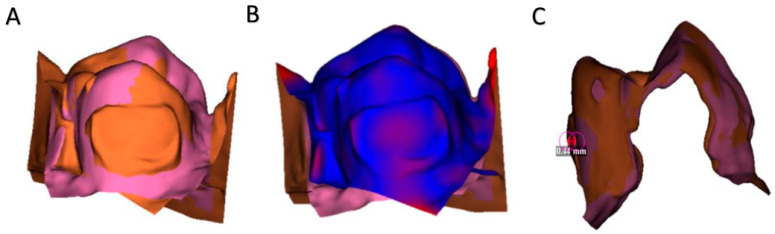
Superimposition methodology for enamel damage and cement remnants evaluation. (**A**). Superimposition performed of STL1-STL2 of a segmented premolar. (**B**). ColorMap tool, in red distances higher than 0.05 mm are shown, and in blue contact between STLs is shown. (**C**). Sagital view of the superimposition and measurement of the cement thickness (perpendicular to the lingual surface.

In order to assess if enamel damage was being caused, STLs before and after the polishing protocol were performed and superimposed in a digital software. The superimposition technique was previously assessed showing reproducibility (Figure 1) [13].

### 2.6. Methodology of Measuring

The polishing time was counted by the second operator with a mobile chronometer (Apple Inc., Cupertino, CA, USA). The chronometer was turned on when the principal operator started the polishing of the dental arch and was turned off after the total polishing. Time recordings data for each dental arch polishing were introduced in an Excel document.

### 2.7. Statistical Analysis

The statistical analysis was performed using the software: SAS v9.4, SAS Institute Inc., Cary, NC, USA. Statistical decisions were made using a significance level of 0.05.

## 3. Results

### 3.1. Turbine Group

#### 3.1.1. Descriptive Analysis

**Figure 2:**
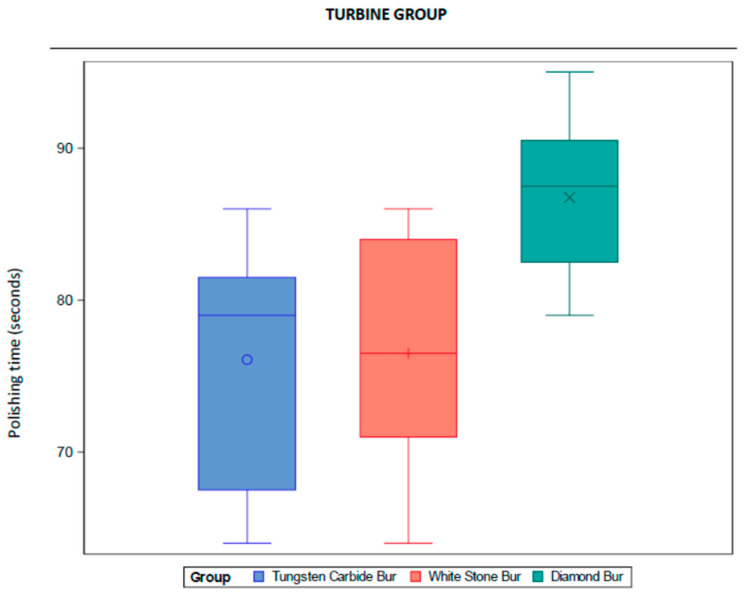
Box diagrams of the descriptive analysis. Turbine group.

The average polishing time was 76.08 s with tungsten carbide bur, 76.50 s with white stone bur and 86.75 s with diamond bur. The descriptive analysis with means, medians, standard deviations, minimums and maximums are shown in Table 1. The information is also represented in a box diagram (Figure 2).

#### 3.1.2. Comparative Analysis

**Figure 3:**
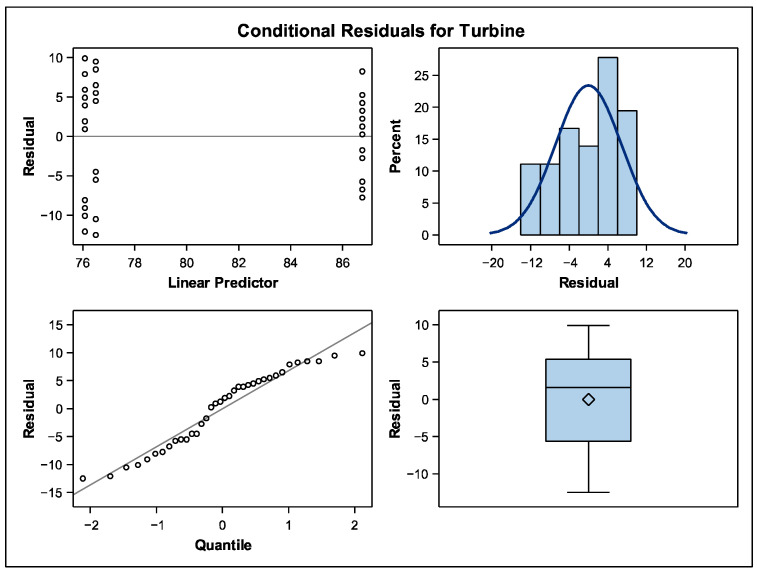
Model validation of the comparative analysis in the turbine group.

The statistical analysis was performed by a lineal mixed model taking into account the group as a fixed factor and the participant variable as a randomized factor. If statistical differences were found, a two-by-two contrast was performed afterwards. In order to correct the Type I error, in the multiple contrast, the *p*-values obtained were corrected by the Tukey–Kramer adjustment model (Table 2 and Table 3). The model validation was also performed by an adjustment graphic analysis Figure 3. The conditional residual statistical model was used in this trial because this statistical analysis provide information in cases in which the operator plays a particular role in the results of the model. This analysis allows the evaluation of unusual patterns that should be taken into account for future research. It provides information on the quality of the adjustment taking into account specific considerations as the operator capability or others. In Figure 3 it is observed in the quartile that the linear predictor and the quantile are stable.

### 3.2. Contraangle Group

#### 3.2.1. Descriptive Analysis

**Figure 4:**
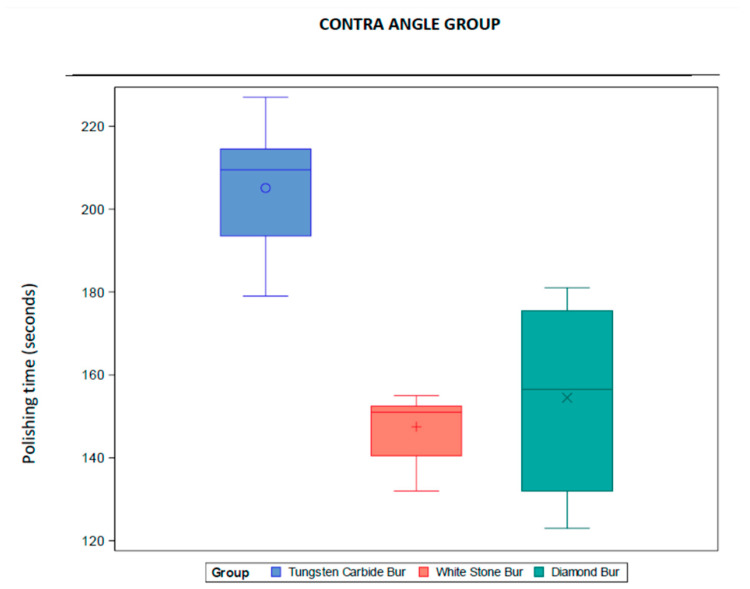
Box diagram of the descriptive information in the contra angle group.

The average polishing time was 147.50 s with white stone bur, 154.50 s with diamond bur, and 205.08 s with tungsten carbide bur. The descriptive information was shown in Table 4. The information was also represented in a box diagram (Figure 4).

#### 3.2.2. Comparative Analysis

**Figure 5:**
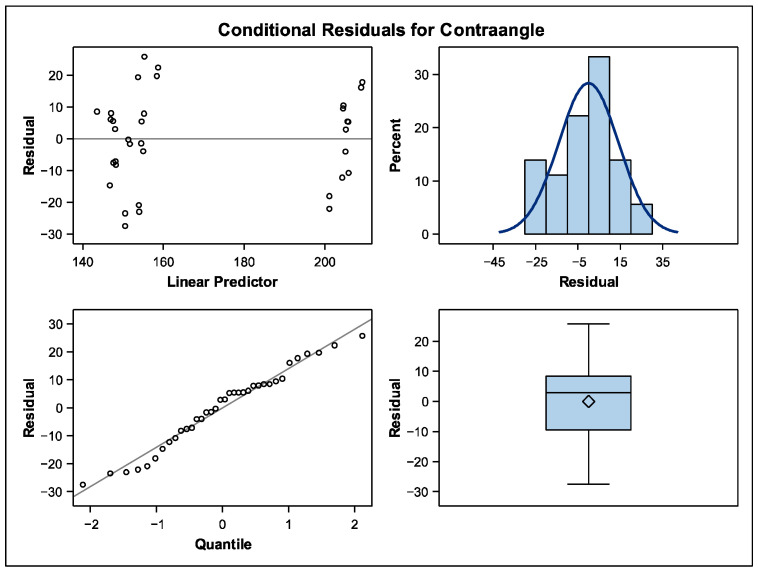
Model validation of the contra angle group.

In the contra angle group, a Type III test of fixed effects was performed in order to correct the *p*-value (Table 5). Statistical differences were found between groups. The tungsten carbide bur wasted more time in comparison with the diamond bur and also the white stone bur in the contra angle group (*p* < 0.005). These results are found in Table 6 and Figure 5. In Figure 5, the conditional residual analysis showed a linear predictor that is less stable but the quantile remains in a stable prediction. This means that the contra angle might be more dependent on the operator technique.

## 4. Discussion

The conclusion of this in vitro study shows that there are differences in polishing velocity patterns between burs and drills in the polishing protocol after resin customized lingual brackets debonding. It seems that tungsten carbide bur is the fastest while using turbine in contrast with the contra angle drill when it is the slowest. This might mean that it would be recommended to use tungsten carbide bur with a turbine and the second step of polishing with another tool if efficiency is required. In the present in vitro study, the results showed that there are statistical differences in polishing efficiency between low- and high-speed handpieces with the same bur. These results showed that the recommended bur for polishing after lingual orthodontic multibracket appliances is different when contra angle or turbine were used. In the results of this trial, the tungsten carbide bur showed statistically better results in terms of polishing efficiency, while white stone bur was preferred when contra angle was used. Neither with low-speed nor high-speed handpiece is the fine diamond bur recommended if efficiency is required for polishing. However, no statistical differences were observed in the contra angle group between white stone and diamond bur. In addition, the polishing protocol with contra angle seemed to be more operator dependent. So, this should be taken into account for future research in order to avoid operator biases. Moreover, the bracket customization and indirect cementation is briefly studied in the literature, and it has special considerations during the adhesion procedure. The customization of this bracket is made by resin added to the mesh looking for the best surface fitting in each surface. That might mean that less cement is needed for the bracket adhesion compared to a conventional bracket adhered on the lingual surface. In addition, this bracket is indirectly cemented because the individualization of the mesh was performed for a specific area of the lingual surface. Lingual orthodontics adhesion is more difficult than vestibular adhesion with conventional brackets due to the lack of contact allowed between the mesh and the enamel surface lingual surfaces. Lingual surfaces have strong differences in convexity patterns between each tooth and even tooth surface changes from convex to concave are noticed. That is the reason why commonly lingual multibrackets appliances are bonded indirectly. In addition, new individualization protocols were created to improve adhesion and also avoid the side effects of lingual forces application. Cement individualization of the mesh is being used and consists of the application of compact resin to the mesh in order to obtain the mesh adapted to each surface. Then, for the bonding protocol, the current cement for orthodontics is applied to the compact cement. So, three adhesion forces are involved (mesh–compact resin, compact resin–current cement, cement–enamel). The adhesion patterns of this methodology of individualization are still unclear and it might be important to know if polishing protocols should be adapted for this technique. In vestibular multibrackets orthodontics, cement individualization is not normally needed because the convexity of the surfaces is smoother and has not much inter nor intravariability.

In addition, the cement used in this trial is a common cement used in orthodontics for bracket bonding. The adhesion was performed by a 3-step adhesion protocol in which surface conditioning, primer, and resin were used. In a recent meta-analysis, it was observed that acid etching protocols for bracket adhesion had superiority over self-etch primers and self-cure resins. The failure rates for self-etch and self-cure resins were three to six times higher than 3-step protocols, whereas self-cure resins were superior to resin-modified glass ionomer for bracket adhesion [14]. In addition, adhesive precoated brackets were compared to operator-coated protocol, but no significant differences in the failure rates were observed [15]. An enamel roughness assessment in dependence of the adhesive protocol was also studied. Enamel roughness was compared with profilometry after bracket debonding, and it was observed that self-cure resins obtained smoother surfaces than light cured in vitro [16]. Moreover, enamel roughness was studied after polishing with different protocols of Sof-Lex systems. It was observed that a tungsten carbide bur mounted on contra angle did not produce significant damage to enamel and no discoloration was observed when Sof-Lex discs or wheels were used [17].

In this in vitro trial, the cement remnants and the enamel loss after debonding customized lingual brackets were studied by the same operator. The volume and area of both variables were measured in order to justify the operator technique. It was measured using a novel digital technique based on standard tessellation language images (STL) superposition called morphometric measuring [13,18]. In addition, a second methodology based on STL superposition was created for the same purpose but using a cephalometric three-dimensional software in order to enhance the orthodontist to provide a better investigation of the polishing technique [19]. Other authors have also studied polishing but have focused on enamel roughness after the polishing protocol. The diamond paste with Soft-Lex discs showed good outcomes in final roughness and it was recommended [20]. Also, it was shown that a two-step or even a three-step polishing protocol allowed a better roughness outcome than a one-step one, showing statistical differences between groups [21]. In further articles, it might be interesting to compare the efficiency of a two-step and three-step protocol taking into account enamel roughness and cement remnants amount in order to stabilize a polishing protocol for lingual orthodontics. Currently, efficiency during polishing has not been widely studied, but a similar article recommended turbine in order to obtain a smoother enamel surface and enhance the efficiency while cutting. Electric handpiece did not show better outcomes than turbine when comparing the roughness variations on enamel [22]. On zirconia surfaces, it was observed that roughness was similar using a zirconia special than a tungsten carbide bur. However, white stone was contraindicated because it showed severe damage [23,24]. In orthodontics, the burs for polishing has also been studied comparing the initial to the final roughness. It has been shown that the composite and the fiber glass burs achieved smoother enamel surfaces than the tungsten carbide bur alone. Enamel roughness remains important in dentistry because it avoids the plaque retention on the surfaces preventing caries [25]. Nowadays, with the emergence of digital dentistry, orthodontics was performed indirectly bonded with the aid of computer-aided technology. The digital planning and indirect bonding were a considerable improvement for lingual orthodontics due to the difficult access [9,10]. In addition, efficiency in orthodontics also means a reduction in the long-term treatment time. Computer-aided/computed-designed (CAD-CAM) bracket showed higher efficiency in chair-time compared to conventional brackets [26]. The brackets used in the presented study were CAD-CAM designed brackets for lingual orthodontics. This system is a lingual straight-wire and self-ligating system in which the brackets are customized by adding composite on the base bracket allow the technique to individualize the case. These brackets have a square slot, which showed statistical accuracy [27]. On the other hand, efficiency and productivity have been studied before, and it has been said that the postgraduate dental education is important for enhancing this variable [28]. Nowadays, the digital approach to dentistry has been very important in terms of efficiency. The digital workflows allow the clinician to reduce times and improve the outcomes in impression and laboratory communication. Also, the patients prefer the digital impressions over conventional impressions. The reductions in time with the three-dimensional approach can reduce the 14 min impression time [29]. Also, it was reported that dentists who provide assistance in community care health centers were less efficient than those who worked privately in Europe. The assistant knowledge is also a very important variable, which can improve the productivity [30]. The presented study was carried out by a three-year specialized orthodontist and an assistant with orthodontics knowledge who recorded the time records.

The limitations of the study are that the trial was in vitro so the real concerns of a lingual multibracket bonding was not assessed. This should include the saliva, the patient comfort, and the difficult access to the lingual surfaces. Moreover, the trial was one-operator made, so the polishing technique is hand-sensitive, and the results might not be representative for all the clinicians. The operator was a lingual technique experienced orthodontist. This is considered a bias in the study. Also, more variables, such as enamel roughness, surface temperature and volume amount of cement remnants would be interesting to evaluate in order to obtain more significant results. In addition, for future studies a two-step protocol and a fiber glass bur might be included to compare the effectiveness of this polishing techniques to assess which is better for enamel roughness variables. In this trial, only three burs were evaluated, but within the results presented, further articles with safer burs and polishing points are considered.

## 5. Conclusions

The fastest bur in the turbine group was tungsten carbide, which was the slowest in the contra angle group. Tungsten carbide bur is recommended for polishing after lingual brackets debonding in order to achieve efficiency.

## Figures and Tables

**Table 1:** Descriptive analysis of the turbine group time expressed in seconds.

Analysis Variable: Turbine							
Group	N	N Miss	Mean	Median	Std Dev	Minimum	Maximum
Tungsten Carbide Bur	12	0	76.08	79.00	7.69	64.00	86.00
White Stone Bur	12	0	76.50	76.50	7.95	64.00	86.00
Diamond Bur	12	0	86.75	87.50	5.05	79.00	95.00

**Table 2:** Test of fixed effects of the turbine group.

Type III Tests of Fixed Effects					
Effect	Num DF	Den DF	*F* Value	*p* Value	
Group	2	22	8.89	0.0015	*

* Statistical differences.

**Table 3:** Comparative analysis between burs in the turbine group.

Differences in Time Least Squares Means Adjustment for Multiple Comparisons: Tukey–Kramer								
Group	Group	Estimate	Standard Error	DF	*t* Value	*p* Value	Adj *p*	
Tungsten Carbide Bur	White Stone Bur	−0.4167	2.8658	22	−0.15	0.8857	0.9884	
Tungsten Carbide Bur	Diamond Bur	−10.6667	2.8658	22	−3.72	0.0012	0.0033	*
White Stone Bur	Diamond Bur	−10.2500	2.8658	22	−3.58	0.0017	0.0046	*

* Statistical differences.

**Table 4:** Descriptive analysis of the contra angle group.

Analysis Variable: Contra angle							
Group	N	N Miss	Mean	Median	Std Dev	Minimum	Maximum
Tungsten Carbide Bur	12	0	205.08	209.50	15.35	179.00	227.00
White Stone Bur	12	0	147.50	151.00	7.28	132.00	155.00
Diamond Bur	12	0	154.50	156.50	21.70	123.00	181.00

**Table 5:** Modelization model of the contra angle group.

Type III Tests of Fixed Effects					
Effect	Num DF	Den DF	*F* Value	*p* Value	
Group	2	22	51.85	<0.0001	*

* Statistical differences.

**Table 6:** Tukey–Kramer adjustment of the differences in means in the contra angle group.

Differences in Time Least Squares Means Adjustment for Multiple Comparisons: Tukey–Kramer								
Group	Group	Estimate	Standard Error	DF	*t* Value	*p* Value	Adj *p*	
Tungsten Carbide Bur	White Stone Bur	57.5833	6.1709	22	9.33	<0.001	<0.001	*
Tungsten Carbide Bur	Diamond Bur	50.5833	6.1709	22	8.20	<0.001	<0.001	*
White Stone Bur	Diamond Bur	−7.0000	6.1709	22	−1.13	0.2689	0.5038	

* Statistical differences.

## Data Availability

The original contributions presented in this study are included in the article. Further inquiries can be directed to the corresponding author.
